# Evaluating artificial intelligence-enabled medical tests in cardiology: Best practice

**DOI:** 10.1016/j.ijcha.2025.101783

**Published:** 2025-08-30

**Authors:** Jonas L. Isaksen, Malene Nørregaard, Martin Manninger, Dobromir Dobrev, Thomas Jespersen, Ben Hermans, Jordi Heijman, Gernot Plank, Daniel Scherr, Thomas Pock, Vajira Thambawita, Michael A. Riegler, Jørgen K. Kanters, Dominik Linz

**Affiliations:** a Laboratory of Experimental Cardiology, University of Copenhagen, Copenhagen, Denmark; b Division of Cardiology, Department of Internal Medicine, Medical University of Graz, Graz, Austria; c Institute of Pharmacology, West German Heart and Vascular Center, University Duisburg-Essen, Essen, Germany; d Department of Medicine and Research Center, Montreal Heart Institute and Université de Montréal, Montreal, Canada; e Department of Integrative Physiology, Baylor College of Medicine, Houston, TX, USA; f Department of Biomedical Sciences, University of Copenhagen, Copenhagen, Denmark; g Department of Cardiology, Cardiovascular Research Institute Maastricht (CARIM), Maastricht University Medical Centre, Maastricht, the Netherlands; h Division of Medical Physics and Biophysics, Gottfried Schatz Research Center, Medical University of Graz, Graz, Austria; i Institute of Computer Graphics and Vision, Graz University of Technology, Graz, Austria; j SimulatMet, Oslo, Norway; k Simular Research Laboratory, Oslo, Norway; l Center for Biosignal Research, University of California, San Francisco, San Francisco, CA, USA

**Keywords:** Artificial Intelligence, Machine Learning, Deep Learning, Medical Test, Evaluation

## Abstract

Machine learning methods are increasingly used in cardiovascular research. In order to highlight opportunities and challenges of the evaluation of studies applying machine learning, we use examples from cardiac electrophysiology, a field characterized by large and often imbalanced amounts of data. We provide recommendations and guidance on evaluating and presenting supervised machine learning studies. We recommend proper cohort selection, keeping training and testing data strictly separate, and comparing results to a reference model without machine learning as basic principles to ensure the quality of studies using machine learning methods. We furthermore recommend specific metrics and plots when reporting on machine learning including on models for multi-channel time series or images. This Best Practice paper represents a possible blueprint to help evaluate machine learning-based medical tests in cardiac electrophysiology and beyond.

## Graphical abstract

**Figure:**
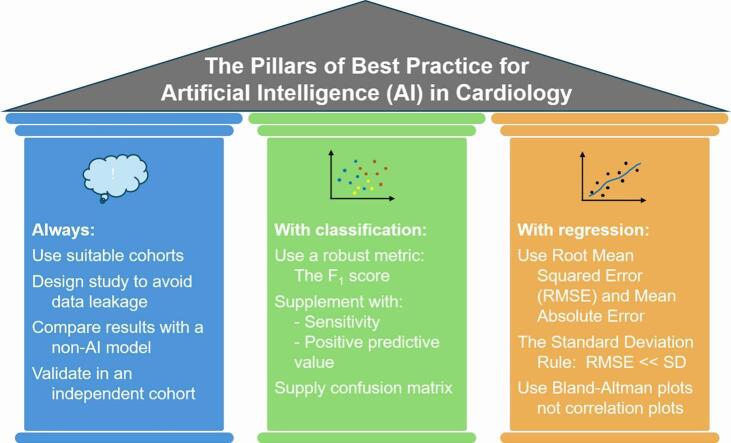

## 1 Scope

Supervised machine learning methods are data-driven models that are trained to make a decision [[1], [2], [3]]. Most often, the problem is formulated as a classification problem with a distinct number of possible answers (typically just yes or no) [4]. In cardiology, machine learning has been widely applied in studies of cardiac electrophysiology. Examples include prediction of stroke based on atrial fibrillation (AF) burden [5], prediction of ventricular tachycardia or fibrillation in patients with cardiomyopathy based on monophasic action potentials [6], AF detection or prediction from electrocardiograms (ECG) [7,8] or photoplethysmograms (PPGs) [9], ECG rhythm classification (multiple categories) [10], and even mortality based on ECG analysis [11]. Less commonly, the problem is formulated as a regression problem, where the results can be any number. Examples include PPG-based estimation of heart rate [12] and ECG-based estimation of age [13].

Machine learning models trained with large data sets are more often susceptible to the pitfalls of *data leakage*, *class imbalance*, and incomplete metric reporting than classical statistical models (see Box 1 for a glossary of commonly used terms in machine learning research). Classical epidemiological fallacies such as immortal time bias, spurious associations, and confounding also still apply, making reporting on machine learning models even more challenging [14]. These pitfalls are important to avoid for all clinical research, whether published in a data science journal or a clinical journal.

Box 1 **Glossary.**

**Attention maps** are explainability methods to explain how a model relates an input to the output by highlighting the relevant parts of the input.

**Data leakage** is the inclusion of different recordings from the same patient in different sets (e.g. training set and testing set). Will lead to overestimated performance.

**Ensemble models** are the combination of predictions from several models.

**Generalization** describes how well a model performs in a new population.

**Hyper parameters** are settings of a model, e.g. the number of trees in a random forest, the learning rate and optimizer for a neural network, or the number of neighbors K used in K nearest neighbor classification.

**Imbalanced classes** are classes that are not evenly distributed. There is no clear-cut definition, but a conservative rule of thumb is that classes are imbalanced if the ratio exceeds 80%/20%.

**K-fold cross-validation** is the repeated training and validation across a training cohort split into K subsets with K models. Enables validation predictions for all patients in the training cohort and produces an estimate of uncertainty.

**Over-fitting** is the phenomenon, where a model, in addition to modelling a desired relation, also models the cohort-specific variation (noise). An over-fitted model will not generalize as well to other populations as a well-fitted model.

**Supervised learning** is model training with known outcomes (labelled data). Unsupervised learning with unlabeled data can be e.g. clustering or compression.

**The Standard Deviation Rule** states that the root mean squared error of a model’s continuous predictions should be much lower than standard deviation of the outcome in the cohort.

**Training, validation, and testing sets** are data from separate patients used to train, optimize, and validate the model, respectively. Data may come from one or more cohorts. “Validation” and “testing” are sometimes used interchangeably.

In this Best Practice paper, we present recommendations and guidance for proper evaluation of supervised machine learning models within cardiology by highlighting some examples from clinical cardiac electrophysiology. The recommendations are based on those applicable to any medical tests with an emphasis on the pitfalls often associated with machine learning.

**Fig. 1:**
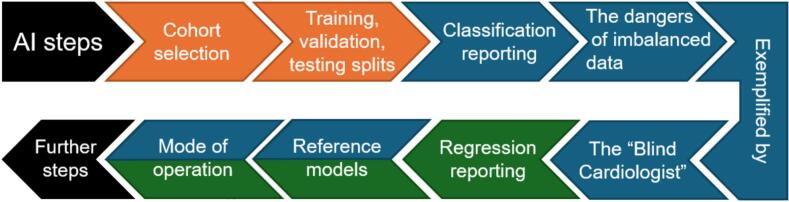
All artificial intelligence (AI) projects start with cohort selection and data splitting (orange). Classification problems (blue) require correct reporting and are often subject to imbalanced data – the dangers of which are exemplified by the “Blind Cardiologist.” Regression problems (green) require different reporting from classification problems. Reference models and mode of operation are important for both types of problems. Further step are required for most AI projects before clinical integration. (For interpretation of the references to colour in this figure legend, the reader is referred to the web version of this article.)

This Best Practice paper outlines design and reporting of both regression and classification problems, including comparison with reference models and analysis of mode of operation. See Fig. 1 for a flow chart.

## 2 Cohort selection, training and testing sets

As with other tests, conclusions on machine learning methods can only be drawn for populations similar to the ones used in the study. It is therefore important that the cohorts used are appropriate, well described (patient characteristics and inclusion criteria) [15] and that the conclusions of the paper align with the study design including choice of cohort.

Compared with classical statistical models, machine learning models have many more parameters to learn, and work with data of much higher dimensionality. Therefore, the number of training samples needed can be much higher for machine learning models than for classical models. Ideally, authors have multiple cohorts available, which can be combined or used separately. In either case, a hold-out *testing set* should be set aside for final testing. Importantly, the model may not be changed after “unblinding” oneself to testing set results, meaning the testing set can only be used once.

The remaining part of the cohorts should be divided into a *training set* and a *validation set,* either with one fixed split or in *K-fold cross-validation*. The latter option 1) enables validation predictions for all patients in the training-validation cohort, 2) produces an estimate of model variation or uncertainty, 3) avoids an “unlucky split”, and 4) allows for the creation of an *ensemble model*, which is typically better than any of the individual models.[16,17].

The validation set (also termed fine-tuning set) is used to optimize the *hyper parameters* of the model or algorithm and thereby model performance. Using the results of the validation set repeatedly to optimize the model is necessary, but inevitably leads to a model that is *over-fitted* to these patients. Thus, the results from the validation set must be assumed overly optimistic about the model’s true performance and the model cannot be expected to *generalize* to unseen patients with these metrics. Therefore, after final optimization, the model is tested in the testing set and this performance reported as the main result.

The testing metrics indicate how the model performs in unseen patients within the same setting. However, differences in equipment, procedures, practices, populations, and noise can lead to poorer generalization.[15,18,19] An example of how a change in population can change machine learning performance, was how an existing sepsis warning system failed in response to the Covid-19 pandemic changing the hospital population.[20,21].

In a setting with more than one record per patient, patients must be completely separated between training, validation, and testing sets, i.e. each patient with all their respective records can only appear in one of those sets. Failure to do so is termed *data leakage* and results in a changed interpretation of the validation and test results. With complete separation, the interpretation is “model performance on unseen, similar patients” however, with data leakage, the interpretation becomes “performance in this cohort only with no promise of generalization” or alternatively “performance in new patients after manual personalization of the algorithm”.[22] In general, we recommend that authors be clear about avoiding data leakage by explicitly stating “we split the data by patient” in the methods.

## 3 Outcomes and “ground truth”

AI models are designed to derive the relation between the input (data) and the output (outcome) as closely as possible. Thus, the operation and result of the AI model will depend on how the output, termed the “ground truth”, was derived. If the outcome was derived using an existing algorithm, the AI model with try to mimic that algorithm.[23] As a rule of thumb, the AI model will never be better than the data-outcome pair it was trained on – with one notable exception: The AI model may not be able to reproduce spurious, complex, and rare mistakes in the outcome.

If the outcome was derived using one or more human experts, the AI model will try to mimic those specific experts. Thus, if there are *systematic* errors or omissions in the outcome associated with the data, the model will systematically reproduce those errors.

The nature of AI models may impose challenges in validating AI models. An AI model trained to identify ventricular tachycardia using an American definition of ≥ 6 consecutive beats[24] will have a low sensitivity if validated on data annotated with the European definition of ≥ 3 consecutive beats.[25] To properly validate and use such an AI model with a different “ground truth”, an adaptation known as fine-tuning or transfer learning is needed.[26].

Not all validation is meaningful. Comparing AI model performance to human experts is only relevant if we currently rely on human experts for that specific task. For instance, arrhythmia classification compared to cardiologists is meaningful,[10] whereas comparing AI and radiologist for the detection of left ventricular structural abnormalities on chest X-rays[27] is less meaningful, when the gold standard is cardiologist interpretation of echocardiograms. In such cases, the absolute performance is more informative than the performance relative to human experts.

## 4 Classification metrics and figures

**Fig. 2:**
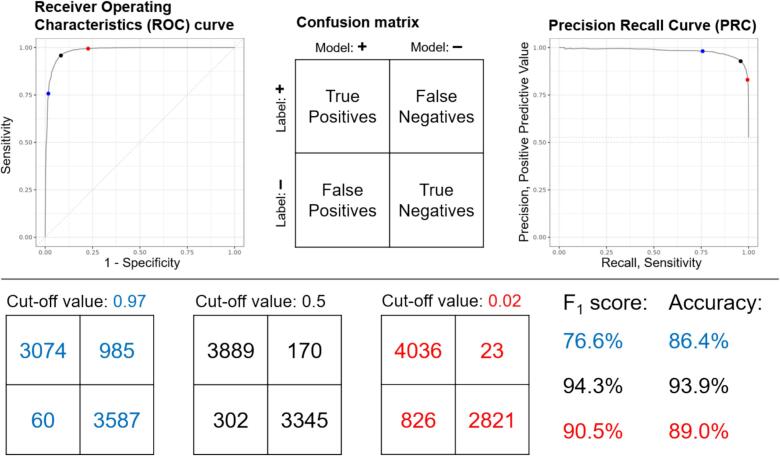
Two curves display possible model performances for different cut-off values when assigning positive and negative labels (top-left: receiver operating characteristic curve based on sensitivity and 1-specificity; top-right: precision-recall curve based on positive predictive value and sensitivity). The model was optimized for a minimal loss for the cut-off value of 0.5 (black). The F_1_ score and accuracy are highest close to this optimum. With a higher cut-off value (0.97, blue), less false detections occur, but also fewer true positives are found leading to a lower sensitivity. However, specificity and positive predictive value are higher. With a lower cut-off (0.02, red), more cases are labelled positive (higher sensitivity) with the opposite effect on positive predictive value and specificity. See Box 2 for metric definitions. (For interpretation of the references to colour in this figure legend, the reader is referred to the web version of this article.)

Reporting of AI studies with one or more classes requires use of suitable metrics and figures. The core of AI performance is the confusion matrix i.e. the cross-tabulation of actual labels with predicted labels. When reporting on classification problems, the confusion matrix should always be presented – either in the primary text or in a supplement (see Fig. 2, top center and bottom row). Additionally, model performance should be quantified with appropriate metrics.[28] Commonly used metrics are presented in Box 2.

Box 2 **Commonly used metrics.**

**Sensitivity** or **recall** is the fraction of true cases that are detected with the test.

**Positive predictive value (PPV)** or **precision** is the fraction of predicted cases that is a true case.

**Specificity** is the fraction of people who were not cases who are predicted non-case.

**Negative predictive value (NPV)** is the fraction of patients predicted negative that did were not a case.

**Accuracy** is the fraction of model predictions that are correct.

**F_1_ score** is the harmonic mean of sensitivity and PPV and the numeric value 0–100% can be thought of similarly to accuracy. The F_1_ score is robust also in case of class imbalance.

**Matthews’ correlation coefficient** is the binary variant of Pearson’s correlation coefficient and takes on values from −1 (perfectly incorrect test) over 0 (random and thus useless test) to 1 (perfect test).

**Root mean squared error (RMSE)** is the average squared distance between predictions and ground truth scaled back to the non-squared level. This metric detects large outliers due to the squared term.

**Mean absolute error (MAE)** is the average numeric error between predictions and the ground truth.

*Sensitivity* and *specificity* are traditionally used to present the performance of a test, but these metrics should not stand alone when classes are *imbalanced*. In medical literature, metrics are most often reported in percent, whereas the data science community often uses a decimal number. Either is acceptable, but metrics should be reported consistently.

We recommend sensitivity and *positive predictive value* (PPV) to characterize model performance, since these metrics are unaffected by a large fraction of true negative predictions. As a single-number metric, we recommend the *F_1_ score*, which is the harmonic mean of sensitivity and PPV, over accuracy, because the F_1_ score is robust also when classes are imbalanced (see section 5: **The problem with imbalanced data**).

*Matthews’ correlation coefficient* is a viable alternative to the F_1_ score and was reported more robust than the F_1_ score when the positive class is much more common than the negative class.[29] However, Matthews’ correlation coefficient only works for two-class problems.

If authors want to present an overall F_1_ score for a multi-class classification model, we recommend using plain averaging without weighting (termed “macro averaging”), because weighting the F_1_ scores by class prevalence reintroduces the class imbalance to the metric that it otherwise avoids.

A machine learning model that discriminates two or more classes typically assigns likelihoods (in percent) to each class, and the predicted class is the most likely class. In the two-class case it is common to show how model performance changes when the cut-off varies from 0 (all tests considered positive) to 1 (no case considered positive) instead of the standard 0.5 cut-off. A change in cut-off value changes predictions and, therefore, how the model operates and thereby the confusion matrix. Such fine-tuning is permitted in the training, but cannot be done after final testing due to potential *over-fitting* to the testing set.

The Receiver-Operator Characteristics (ROC) curve displays the sensitivity against (reversed) specificity and the precision-recall curve (PRC) displays sensitivity against positive predictive value when the cut-off value is varied (Fig. 2). Because the ROC curve is built on sensitivity and specificity, the curve is less useful when classes are not balanced. The PRC, however, is robust to class imbalances,[2] because sensitivity and PPV both avoid counting the heavily overrepresented True Negative cell (Fig. 2).

The area under the ROC curve is numerically identical to the concordance index (c-index). The c-index corresponds to the likelihood across all patients that the model will assign a higher likelihood to a case than to a control, which is an estimate of how well the model will perform for an individual. Thus, the worst value of the c-index is 0.5 (50 %, dotted line on Fig. 2, top left). Because it is a measurement across all patients, the area under the ROC curve becomes less reliable as classes become increasingly imbalanced.[30].

We are not aware of any similar interpretation of the area under the PRC. The area under the PRC for a model that does not perform well (and for one that does) depends on the prevalence, since a model that predicts only positives (only left column of confusions matrices on Fig. 2, resulting in 100 % sensitivity) will have a PPV corresponding to the prevalence (dotted line on Fig. 2, top right).

We recommend that area metrics do not stand alone. In addition to the weaknesses outlined above, the areas do not represent the model in action well. When a model is deployed, it will have a fixed operating point, corresponding to a single, predefined point on the curves. Sensitivity, PPV, and F_1_ score describe this operating point and should always be reported, because these metrics help show how the model works in the individual case. However, the figures discussed and the associated areas may be useful for comparing several models on the same population.

It is important to note that good metrics can never ensure that a model never makes mistakes in individual cases. Indeed, AI models may rarely make obvious mistakes in cases that appear straight-forward.[31] For instance, a model may predict normal sinus rhythm for a specific ECG although the rhythm is clearly ventricular fibrillation. These obvious mistakes are known as AI hallucinations or AI confabulations.[[32], [33], [34]] Such mistakes can be lethal for the patient, and may reduce clinician trust in AI models. This challenge currently limits the use of AI models without human overview.[32].

## 5 The problem with imbalanced data

**Fig. 3:**
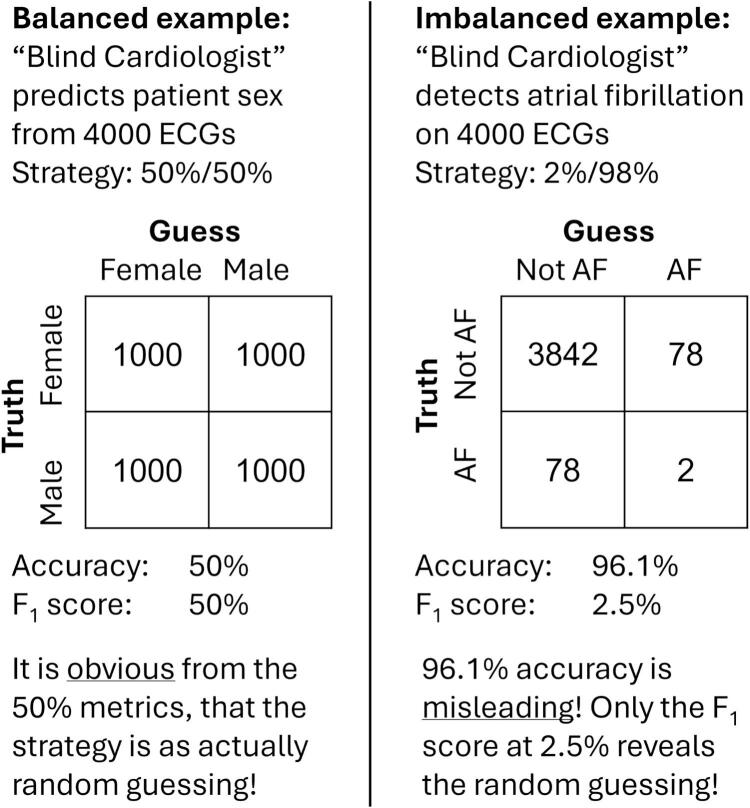
The “Blind Cardiologist” solves a balanced and an imbalanced classification problem. In both cases, the strategy is random guessing with a likelihood that matches the expected prevalence. In the balanced case (*left*) the metrics reveal the strategy as random guessing, however in the imbalanced case (*right*) the accuracy is misleadingly high due to the rare prevalence of the disease in the population (atrial fibrillation, AF, set to 2%).

Imbalanced classes introduce challenges as described above, because the majority class is weighted much higher than the minority class(es).[2] The hypothetical “Blind Cardiologist” in Fig. 3 illustrates this example:[35].

If the “Blind Cardiologist” were to assess patient sex based on an ECG, they would have approximately a 50 % chance of guessing correctly and an accuracy of 50 %. This accuracy is obviously not better than chance because the distribution of sexes is balanced. The F_1_ score is also 50 %.

However, in a screening of random individuals for AF, the “Blind Cardiologist” will have to read ECGs without eyesight. Because the “Blind Cardiologist” knows that the prevalence of AF is around 2 % in the general population, they will predict 2 % at random to have AF and get an accuracy of 96.1 %. The F_1_ score, however, is 2.5 %, revealing the poor performance.

Likely, the “Blind Cardiologist” is smarter than this. They could optimize their accuracy by only guessing “not AF,” but then their sensitivity (and F_1_ score) would drop to 0 %. More likely, they would interview the participants for risk factors and possibly get an accuracy above 98 %. The F_1_ score would scale with how well the risk factors predict AF, but it would once again more accurately reflect the performance than the accuracy metric. Whereas a high accuracy is no guarantee for a good model, a high F_1_ score does mean that the model works well irrespective of class imbalances, which means that the model is likely to work in most individual cases, too, and not only in the larger population.

Classes may be considered imbalanced when their distribution distorts the metrics.[35] As a rule of thumb, a distribution more skewed than 80 %/20 % can be considered imbalanced.

Since imbalanced classes may make model training difficult, various strategies, including down-sampling, up-sampling, weighting, and data augmentation, are commonly employed to make classes more balanced. We acknowledge the need for such strategies in training the model, but under no circumstances should these alterations to the population and data be applied to the testing set, because they invalidate the model testing and the subsequent interpretation. Model weighting with unaltered data remains unbiased, as long as the model is not recalibrated in the evaluation.

## 6 Regression metrics and figures

Using AI to predict a number is a regression problem and the reporting of regression studies require suitable metrics and plots. The key metric for regression problems is the root mean squared error (RMSE). Note, that AI models are often trained using the mean squared error (MSE) as the loss function, however, the RMSE is better suited for reporting, because the error is on the same scale as the variable and not on a squared scale. The mean absolute error (MAE) also operates on the same scale as RMSE, but is less sensitive to outliers compared with RMSE due to the squared difference term. Both RMSE and MAE should be reported without normalization.

**Fig. 4:**
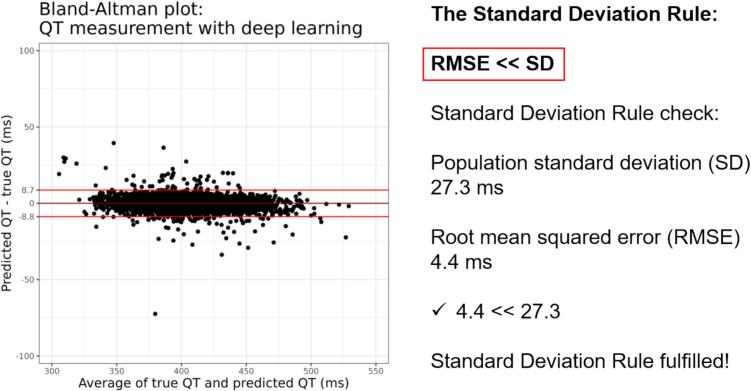
Bland-Altman plot and The Standard Deviation Rule. Left: Bland-Altman plot with indicated bias (0 ms) and limits of agreement (−8.8 ms to 8.7 ms). Right: The Standard Deviation Rule is fulfilled because the root mean squared error of 4.4 ms is much lower than the population standard deviation of 27.3 ms. Data from Hicks et al.[23].

*The Standard Deviation Rule* states that the RMSE of a model should be much smaller than the standard deviation of the outcome of interest (see Fig. 4, right). The Standard Deviation Rule thus gives a measure of comparison for the RMSE metric, because the standard deviation of a marker corresponds to a constant guess on the average across the population.

The correlation coefficient (R) between predictions and true values should not be used to assess how well a model mimics the ground truth values, particularly if these are assessments with error.[36] Notably, correlation is not the same as agreement and cannot separate error from bias.

Instead, we recommend presenting results using Bland-Altman plots.[37] The visual interpretation is straightforward and the limits of agreement can be seen in the light of what is clinically acceptable (see Fig. 4, left). Furthermore, the method allows for assessment of outliers in terms of either measurement error or imbalanced data. This figure thus shows population average performance and individual patient performance simultaneously.

RMSE is a suitable metric for single numbers only, but not for comparing time series as is done in ECG reconstruction and digitization.[[38], [39], [40]] The reason is that the noise level is unknown, and it becomes impossible to discern how large a part error and noise, respectively, contribute to the RMSE. In other words, no one can know what a good RMSE value is for any study. Instead, carefully selected markers should be evaluated with Bland-Altman plots (Fig. 4).[41] This process ensures evaluation of the reconstruction through clinically relevant markers in a way that is not masked by the inherent noise in the recording.

## 7 Reference models

Machine learning models constitute additional complexity compared to traditional statistical tests and many machine learning models are not transparent. Therefore, a minimum requirement should be that machine learning models contribute additional performance compared to traditional methods. This is easily doable with feature-based machine learning models: the reference models should be supplied the same features from the same patients as the machine learning models.

The reference test for a binary classification should be logistic regression, which produces odds ratios (and p-values) that directly link risk factors with odd ratios for the outcome of interest. The reference model for any time-to-event data is a suitable model for survival analysis, for instance Cox regression or Poisson regression. Linear regression is the reference test for a continuous outcome and provides beta coefficients (and p-values) to relate risk factors to the outcome of interest.

Neural networks may work directly on full waveforms of electrophysiological signals and perform feature extraction, processing, and classification in one go.[23] For these models, the same features cannot be supplied to the machine learning model and the reference model. In such cases, we recommend to use common and readily available markers as the reference. A neural network that predicts mortality on ECGs should be compared to a Cox regression model using known ECG risk factors for mortality (e.g. heart rate,[42] QT interval,[43] and T-wave morphology[44]) in addition to sex, age, and other factors important for survival that are encoded in the ECG.[13].

In cardiovascular research, neural networks are often applied to multi-channel time series or images. Here, reference models may also include existing, published models or frameworks, such as ROCKET or InceptionTime for time series.[45,46].

When relevant, a reference model may also be an established clinical score. When Khurshid and colleagues tested their model for AF risk prediction, they compared it to the three established scores: CHARGE-AF, C_2_HEST, and CHA_2_DS_2_-VASc.[47].

## 8 Mode of operation

Traditional statistical models are typically presented with effect estimates (beta coefficients, odds ratios, hazard ratios) for each known variable, making it straightforward to assess what biomarkers or features are important for a given problem.[[48], [49], [50]] Common machine learning practices may obscure the relationship between a biomarker and the outcome, and we present current solutions to these problems.

Normalization is a common practice that can improve model training,[51] but it has the important downside that the parameter scale is changed. For instance, after normalization blood pressure is no longer measured in mmHg but instead on another scale. Solutions may include not normalizing and re-scaling the results back to the original scale (e.g. mmHg).

Principal component analysis is a method for dimensionality reduction, whereby multiple biomarkers are reduced to fewer principal components.[52] To achieve transparent results with models using principal components, the transformation matrix should be provided (possibly as supplementary material). In addition, authors should describe in the text key biomarker contributors to the principal components that were strongly associated with the outcome.

Signal processing with machine learning utilizes known, crafted features. Many machine learning methods have variable importance metrics, including random forest,[53,54] K nearest neighbors,[55] and support vector machines.[56] Many of these methods are based on shapley values and can be directly applied to the models.[57] Thus, the link from input (biomarkers) to predictions are often known in machine learning.

Neural networks (deep learning) – in contrast to other machine learning methods – can operate on raw data and perform feature extraction and analysis in one go.[58] This makes neural networks extremely flexible and powerful, but also makes the mode of operation hidden (a so-called “black box”), because the features use by the algorithm are not known and not available.[[58], [59], [60]] The most promising solution is *attention maps*, which can help identify novel biomarkers based on deep learning models.[23] There are many attention map methods and not one stands out as a de facto standard.[61] A supplementary method is the ablation technique (blanking), whereby parts of an input is masked to assess the impact on model predictions and infer mode of operation.[23].

## 9 Further testing

Developing and validating a machine learning model is just a first step. Authors should remember that such a model is still a long way from implementation.[62,63] Next steps may be a prospective validation with or without silent implementation, before the model is formally validated in a randomized controlled trial to study of the consequences of implementing the model.

Proper validation of suggested medical tests is a hallmark of modern medicine. The CHADS_2_ score was introduced in 2001.[64] In the 2006 ACC/AHA/ESC guidelines for the management of patients with AF,[65] the CHADS_2_ score was quoted, but the score was not recommended to guide treatment. In 2010, the score was amended and renamed CHA_2_DS_2_-VASc,[66] but the 2011 guidelines[67] still did not recommend the use of the score to guide treatment. However, following extensive validation,[68,69] the 2014 guidelines recommended individualized anticoagulation based on the CHA_2_DS_2_-VASc score,[70] with a 2024 update to the score to CHA_2_DS_2_-VA (without sex) because sex was *de facto* ignored in practice.[71].

Machine learning may be a rapidly growing field within cardiology, but clinical validation of these methods remains important and remains a time-consuming process.[72] The AI-ECG algorithm for detection of AF during periods of sinus rhythm was first presented by the Mayo clinic in 2019.[73] In that paper, they present a sensitivity of 79.0 % and a specificity of 79.5 %. Although not presented, one can calculate the PPV to 26.1 %. They then tested their algorithm in a new population of patients without known AF and with embolic stroke of unknown source with almost similar results (sensitivity: 63 %, PPV: of 23 %). In the Batch Enrollment for AI-Guided Intervention to Lower Neurologic Events in Unrecognized AF (BEAGLE) study,[74] they tested the algorithm prospectively, and found that algorithm-predicted high-risk individuals more often developed AF,[75] but it was not reported whether use of the algorithm could in fact lower neurological events as the title indicated. Therefore, a new study is currently on-going to assess long-term outcomes of using the algorithm compared to control (NCT05923359). This example shows that also within the field of machine learning, the path from discovery to implementation is long.

Importantly, the wording used when reporting on machine learning results should reflect the validation stage of the proposed model bearing in mind that models are not implemented in clinical practice without thorough prospective validation.[76].

## 10 Best practices

Our recommendations for reporting on machine learning models in cardiovascular research can be summarized as follows (see also the **Graphical Abstract**):

Always:

- Use suitable cohorts and sufficient data for your problem, ideally more than one cohort.
- Avoid data leakage
- Compare results to suitable reference models, including one without machine learning
- If possible, validate your model in another independent cohort to test generalization

For classification problems:

- Use a robust overall metric such as the F_1_ score. Accuracy is only useful if classes are somewhat balanced.
- Supplement the overall metric with at least sensitivity and positive predictive value.
- Provide the full confusion matrix in the publication or as a supplement.

For regression problems:

- The standard deviation rule: root mean squared error should be much lower than the outcome standard deviation.
- Root mean squared error may be supplemented with other metrics such as the mean absolute error.
- Use Bland-Altman plots instead of correlation plots.

Grant support:

JLI is supported by a grant from the Danish Cardiovascular Academy (PD2Y-2023004-DCA). The Danish Cardiovascular Academy is funded by the Novo Nordisk Foundation and the Danish Heart Foundation, grant number NNF20SA0067242. MN is supported by a grant from the Danish Data Science Academy and the Danish Cardiovascular Academy (PhD2024015-DCA-DDSA). The Danish Data Science Academy is funded by the Novo Nordisk Foundation (NNF21SA0069429) and the VILLUM FONDEN (40516). This work was supported by a research grant from the Novo Nordisk Foundation Young Investigator Awards 2021 (NNF21OC0066480) to DL. This project is supported by the Innovative Health Initiative Joint Undertaking (IHI JU) under grant agreement No 101172997. The JU receives support from the European Union’s Horizon Europe research and innovation programme and COCIR, EFPIA, Europa Bío, MedTech Europe, and Vaccines Europe. DD is supported by grants from the Deutsche Forschungsgemeinschaft (Research Training Group 2989, project 517043330), National Institutes of Health (RO1HL131517, RO1HL136389, RO1HL163277, RO1HL160992, RO1HL165704, RO1HL164838, and RO1HL176651), and the European Union (large-scale network project MAESTRIA No. 965286). JH is supported by the Netherlands Organization for Scientific Research (NWO/ZonMW Vidi 09150171910029) and the Dutch Heart Foundation (grant number 01–002-2022–0118, EmbRACE consortium). TP and GP are supported by the Austrian Science Fund FWF (Grant 10.55776/I6540).

## CRediT authorship contribution statement

**Jonas L. Isaksen:** Writing – review & editing. **Malene Nørregaard:** Writing – review & editing. **Martin Manninger:** Writing – review & editing. **Dobromir Dobrev:** Writing – review & editing. **Thomas Jespersen:** Writing – review & editing. **Ben Hermans:** Writing – review & editing. **Jordi Heijman:** Writing – review & editing. **Gernot Plank:** Writing – review & editing. **Daniel Scherr:** Writing – review & editing. **Thomas Pock:** Writing – review & editing. **Vajira Thambawita:** Writing – review & editing. **Michael A. Riegler:** Writing – review & editing. **Jørgen K. Kanters:** Writing – original draft, Conceptualization. **Dominik Linz:** Writing – original draft, Conceptualization.

## Declaration of competing interest

The authors declare that they have no known competing financial interests or personal relationships that could have appeared to influence the work reported in this paper.
